# A qualitative evaluation of a student midwife placement teaching English to speakers of other languages (ESOL)

**DOI:** 10.18332/ejm/188531

**Published:** 2024-06-13

**Authors:** Clare Maxwell, Amanda Robinson, Pamela Donaghy-Binks, Valerie Fleming

**Affiliations:** 1 Faculty of Health, School of Public and Allied Health, Liverpool John Moores University, Liverpool, United Kingdom; 2 Faculty of Health, Social Care and Medicine, Edge Hill University, Ormskirk, Lancashire, United Kingdom

**Keywords:** student midwife, remo, pre-registration education, ESOL, non-traditional placements, midwifery education

## Abstract

**INTRODUCTION:**

A shortage of UK midwives has put pressure on clinical placements and supervision of student midwives. Alternative placement solutions are needed to provide students with meaningful learning experiences. One such learning experience was a placement undertaken by student midwives who attended a program teaching English to speakers of other languages (ESOL). This study evaluated the impact of the placement on student midwife learning and experiences of the ESOL participants.

**METHODS:**

The 2022 study employed a qualitative design using Kolb’s model of experiential learning as a framework. Ten student midwives placed with the ESOL program and three women enrolled in the program participated. Data were collected via online focus groups with the student midwives and a face-to-face focus group with the women. Data were analyzed using thematic analysis and Kolb’s model of experiential learning.

**RESULTS:**

Four themes were constructed: ‘Putting the scripts aside: expectations versus the reality of being an educator’, ‘Adapting and personalizing teaching’, ‘We are learning too: an environment for mutual learning’, and ‘Taking our learning forwards’. Students faced barriers during their placement and had to adapt their teaching accordingly. They gained crucial knowledge of the challenges faced by women who speak other languages. The women valued the students’ input and together they forged a reciprocal learning environment.

**CONCLUSIONS:**

This study demonstrates how placing student midwives in a unique non-maternity setting has benefits for student learning which are transferrable to future practice. Importantly, it confirms that quality of learning during a novel placement is not compromised for students or participants.

## INTRODUCTION

According to The State of the World’s Midwifery 2021 report^1^, 900000 more midwives are needed globally. From a UK perspective, the Maternity Workforce Strategy recommends a 25% increase in new training placements to meet the projected future shortfall of qualified midwives^2^. However, increasing student numbers requires a corresponding increase in clinical places and midwifery supervisors, the latter of whom there is a current shortage in the UK^3^. To counter this ‘impasse’, Health Education England (HEE) has sought alternative solutions to provide healthcare students with meaningful learning experiences through a Clinical Placement Expansion Program (CPEP). For student midwives, this enhanced offer recognizes opportunities for learning in non-traditional community placements. One such placement was with the ESOL (English to speakers of other languages) program, where student midwives had the opportunity to teach English to women who had difficulty speaking and/or reading the language.

This study aimed to evaluate the impact of the placement on student midwife learning and the experiences of the ESOL program participants. The study objectives were to ascertain student motivations for undertaking the placement, to explore student experiences of undertaking the placement, to explore ESOL participant experiences of being taught by student midwives, and to determine student learning from the placement.

In 2019, the NHS long-term plan^4^ committed to increasing nursing and midwifery training places by 25%, with some 5000 extra places for student midwives. For some UK Higher Education Institutes (HEI), student numbers would increase by up to 50%^2^. Although the latest figures show that applications to nursing and midwifery places were down in the previous year, they continue to exceed the number of training places available^5^. Attracting applicants to midwifery programs is not a problem; however, procuring increased numbers of clinical placements with resultant clinical supervision is a challenge. This is not a new situation, as highlighted by Magnusson et al.^6^ who describe university bureaucracy, lack of accessibility to new placement areas, and a lack of mentors as barriers to placement expansion. They also discuss a further area of concern, which is ‘quality versus quantity’, with increases in student numbers being seen to have the capacity to erode the standard of clinical learning^6^.

To meet the demand for clinical supervision of students in practice, the Nursing and Midwifery Council (NMC) adopted a more flexible model in 2018. Standards for Student Supervision and Assessment (SSSA) allows students to be supervised by different ‘practice supervisors’ rather than being aligned to one mentor, with a ‘practice assessor’ having oversight of student learning^7^. Since the introduction of SSSA, variations of the model have been further piloted, including collaborative learning in practice (CLiP), whereby a group of students are under the supervision of one clinician^8,9^.

Further changes to clinical practice placements have come in the guise of ‘simulation’, with up to 600 hours of non-placement simulation being able to be counted as clinical practice hours for nursing students^10^. This does not apply to student midwives; however, a recent independent and stakeholder review of minimum standards for nursing and midwifery training is calling for more research around simulation and midwifery training to be undertaken before any changes are considered^11^.

Alongside changes to supervision, a widening of the type of placements nursing and midwifery students can undertake has been gathering momentum, most recently subscribed to by the NMC^11^, which in its latest changes to undergraduate midwifery have included a requirement for student midwives to experience placements with alternative maternity providers. In 2013, the Institute of Health Equity recommended that all health professional education programs include meaningful community-based learning to improve student understanding of the wider social determinants of health^12^. This emphasis on skills underpinned by a socio-cultural rather than clinical element was seen as a green light to ‘think outside the box’ in terms of placements for health students^13^. It was also a springboard for a clinical placement expansion program, where £10 million was on offer to clinical providers/placement organizers to support placement growth in midwifery, nursing, and allied health professional placements. This article focuses on an evaluation of the placement in terms of student midwife learning and the experiences of the ESOL participants.

## METHODS

### Study design

A qualitative study design was employed to capture rich data from both the student and participant experience. This aligned with the small numbers participating in the placement and gave opportunities for student and program participant experiences to be explored in-depth. To evaluate the learning that took place, Kolb’s model of experiential learning was selected as a framework to guide the design and analysis of the evaluation^14^. The model was first described by Kolb in 1984 as a process of learning based upon an experience that encompasses concrete experience, reflective observation, abstract conceptualization, and active experimentation. Since then, there have been various iterations of his model^15^, all fundamentally based on Kolb’s original concept. The cycle has suffered an amount of criticism over the years, mainly stemming from its lack of underpinning empirical theory^16,17^. However, it continues to provide a sound basis as a model of learning by experience. In the case of this study, it was especially useful, owing to the placement being an ‘unknown entity’ in terms of potential learning and one that was situated in a concrete experience.

### ESOL program placement

ESOL Stepping Stones ©^18^ is an innovative course designed to deliver basic English language provision to mothers new to English in community settings. It is offered to vulnerable women and their babies in local communities. Evidence shows that migrants and ethnic minority populations have greater difficulty in obtaining, understanding, and acting on information given by health professionals compared to other population groups^19^. To improve the health literacy of women from ethnic minority groups who have difficulty reading or speaking English, a 12-week ESOL program named Stepping Stones© was delivered in the North West of England by the Workers’ Educational Association (WEA) and Integrated Care Board. The objective of the ESOL program was to give women functional language skills and information on health and parenting. This, in turn, has the potential to reduce health inequalities, support access to the healthcare landscape, and improve the health outcomes for this vulnerable population. Ten women had signed up for the program in the North West of England.

Funding was secured from HEE to expand clinical placements for student midwives. A lecturer in practice placements who was also a registered midwife was appointed and scoped out the ESOL program as a placement that student midwives could attend. The overarching aim of the placement was to provide student midwives with a non-traditional community placement, allowing them a unique opportunity to increase their understanding of the challenges faced by those who do not speak English.

Ten student placements were available. All students in year one of their undergraduate midwifery training program at a University in the North West of England were invited to send an expression of interest to the lecturer in practice placements if they wished to be considered for the ESOL program placement. Thirteen students applied, and ten names were randomly selected.

The placement took place over two weeks, and the students attended five face-to-face sessions with the women at the program base in the North West of England. Prior to the placement, the students and lecturer in practice placements worked in partnership to produce images/fact sheets to improve health literacy relating to pregnancy and basic health needs in anticipation of using with the ESOL participants. During the placement sessions, the student midwives were supervised by the lecturer in practice learning, with the students working in pairs and in partnership with the women to improve their English language skills through role-play scenarios. A translator was also available to support the students and women, and their services were utilized throughout. Students used the rest of the time during the two weeks to research, source, and develop further resources for the women.

### Study recruitment and participants

Students undertaking the ESOL program placement were emailed participation information by the research team, detailing an evaluation study of their placement and were contacted one week later to see if they would like to participate. All 10 students consented to take part. Written consent was obtained at first point of data collection.

A research assistant who spoke Arabic attended a series of dates during the ESOL program to discuss the study and recruit ESOL participants who wished to take part. Out of the ten women enrolled on the ESOL program, three mothers agreed to take part in the research study and consented verbally at the first point of data collection.

### Data collection

Student data were collected via three online focus groups using MS teams undertaken by an experienced qualitative researcher. A focus group interview schedule was developed by the research team based on the aims and objectives of the evaluation, which were guided by Kolb’s experiential learning cycle. Questions to students included: ‘What are your motivations for undertaking this placement?’, ‘What are you hoping to gain from the placement?’, ‘Can you describe your experiences of the placement so far?’, and ‘How has the placement impacted your knowledge?’. To capture the whole cycle of experiential learning, focus groups were scheduled pre-placement (1 week prior), mid-placement, and post-placement (1 week after). The focus groups lasted between 30 and 60 minutes and were recorded via MS Teams. In this study, verbatim student comments are referred to as SM (student midwife).

One audio-recorded face-to-face focus group lasting approximately 30 minutes was undertaken with three women on the last day of the student placement by the Arabic-speaking research assistant. Questions were asked about the women’s experiences of being taught by the student midwives to establish the efficacy of the teaching and how receptive the women were to being taught by the students. Three mothers attended this group, and their verbatim comments were identified as EP (ESOL participant).

### Data analysis

The audio focus group was transcribed, and the MS team’s transcriptions were ‘cleaned’. All data were added to a Word document and analyzed using a reflexive thematic analysis as described by Braun and Clarke^20^. The process was both deductive in responding to the aims and objectives of the evaluation and inductive in that data outside the aims and objectives were included in the analysis. When undertaking the thematic analysis, Kolb’s experiential learning model was referred to. The constructed codes and themes were explored using the four elements of the learning cycle to draw meaning from them in terms of student and participant learning.

## RESULTS

This study aimed to assess the value of a student midwife placement in the community-based ESOL program. All of the student midwives were female, aged 19–35 years, and the majority (9/10) identified as White British. The students were at the end of the first year of their midwifery undergraduate training and, as such, had undertaken approximately ≥700 hours of clinical practice across various maternity-orientated hospital and community placements.

The women were all of North African descent, were mothers, and were either asylum seekers or refugees living in a city within the North West of England with various durations of residency in the UK. They had varying levels of English (speaking, reading and understanding) and Arabic was their first language.

Four major themes were generated from the findings of the four focus groups: 1) Putting the scripts aside: expectations versus the reality of being an educator, 2) Adapting and personalizing teaching, 3) We are learning too: an environment for mutual learning, and 4) Taking our learning forwards.

### Putting the scripts aside: expectations versus the reality of being an educator

The students reflected upon their experiences of caring for women and families whose first language was not English within a clinical setting, which was inextricably linked to their motivations to undertake the ESOL placement. These motivations were exclusively centered around the benefits to the women they would be engaging with:

*‘… our trust (Hospital) is so diverse, and I think it’s just important that we’re able to communicate and be accessible for everybody. So it’s just a new skill for us to use.’* (SM)

*‘Well, having been in situations coming across lots of women who have little to no English and seeing on occasions where they have had quite good experience with the service and occasions where they’ve had a poor experience. So I like the idea of being able to… sort of go into the community and sort of reach these women.’* (SM)

The students were enthusiastic about being able to make a change in the lives of the women they would be working with. However, once they commenced the placement, it became apparent that none of the women was pregnant, which the students and the lecturer in placement support had been told to expect:

*‘We had all these scenarios ready to use with pregnant women, and then we found out none of them were pregnant after all!’* (SM)

In addition, the women had very wide variations in their existing English language skills:

*‘Her English was really, really limited, so we kind of almost put the scripts aside because we realized that it just wouldn’t get anywhere.’* (SM)

The students quickly had to ‘re-set’ their original expectations of the work they would be undertaking during the placement. Further challenges came with the concept of ‘role play’, which was the original educational tool the student would use with the women. The women did not understand the abstract construct of role play, and the students, in turn, found it difficult to explain due to the language barrier:

*‘My lady preferred to speak about her previous children, their needs, and their birth. She didn’t understand that it was role play, and then it became confusing because she has a medical condition, and I think she thought I was going to solve that for her.’* (SM)

The students recognized that the original program format needed to be altered if it was to have the positive impact it set out to have. They overcame the confusion around role play by reverting to asking the women general and ‘meaningful’ questions:

*‘We tried anything, it took us about 5 minutes to try and ask her to pretend, and she couldn’t understand 'pretend'. It was easier for us to just ask general questions about when you go shopping and general stuff, such as asking her if she knew any colors, etc.’* (SM)

The reality of the complexities of learning English from the women’s perspective was brought home to the students by one of the women:

*‘So she actually spoke fluently in Arabic and then she said she was basically trying to say to us that’s what it’s like for her the other way around … it is really, really difficult. And it really put it into perspective, cos we don’t know what you’re saying.’* (SM)

This was a powerful tool used by the woman, which provided the students with an understanding of the challenges faced by the women and those they would face themselves as educators.

### Adapting and personalizing teaching

The students worked in pairs with the same woman for five teaching sessions. This provided an element of continuity, which the students recognized was critical to them building a rapport with the women. This rapport was important to the teaching and learning, and in some cases where the women lacked confidence in themselves to verbalize their knowledge of English:

*‘She was really anxious about it all, and she didn't have any confidence in her ability to speak English. She could speak really, really, really limited, basic English, but she couldn’t find the confidence to say it out loud. So, I think she often knew the answers and knew what she wanted to say, but she just couldn’t do it. So, we spent the first 15–20 minutes just trying to make her feel comfortable.’* (SM)

In some cases, the students reciprocated personal information with the women to enhance their relationship; this underpinned their role as educators and was viewed as integral to facilitating the women’s progress:

*‘And she showed us pictures and we showed the pictures of our kids. So, we definitely built up a relationship with her which we took forward into the next week.’* (SM)

For each woman, the students learnt to gauge the individual level of understanding they possessed and were able to respond to this. In some cases, the original role play idea was retained as the central learning tool used by the students:

*‘The woman I was working with played along with the scenario [of being pregnant] very well. She was making stuff up from her own experience in her own country as well.’* (SM)

However, for most women, the students abandoned the scripted roleplays, replacing them with props and images as learning aids and tailoring them to the women they were working with. The students displayed a level of ingenuity and creativity, working to produce resources prior to their next teaching session. In essence, the students had to improvise between teaching sessions. This proved to be successful with many of the women appearing to find the visual aids more beneficial than just words, which they could not read or understand:

*‘We brought some props today and the lady could say what the objects were … you can see she’s wants to show you what she’s saying … you can see, the concentration when she’s trying to tell you what the object is. And she enjoyed it probably just as much as us.’* (SM)

### We are learning too: an environment for mutual learning

The placement focus was to participate in ESOL sessions for women and this was reflected upon by the women themselves, who commented on what they felt they had learnt from the student midwives. This encompassed not only English language skills acquisition but also listening skills, pronunciation, education and general knowledge:

*‘What I know as “pre-eclampsia” I already knew what it meant but the midwives explained it in a different way.’* (EP)

*‘Ethnicity or the country where you’re born. I knew of the expression, but I never knew what ethnicity meant. Or words like job and employment for example. I knew what job was and used it in everyday life, but on forms and such you come across the word employment more and I learned that.’* (EP)

*‘I learned how to set up an appointment at the GP and not only how to make one for the same day but for upcoming days or weeks. I learned how to make an appointment for next week.’* (EP)

*‘Here, they explain in simple ways. This is the first time I actually learned some useful English … We learned things related to everyday life like at the house and shopping and at school and at work and at the hospital.’* (EP)

The placement also provided learning opportunities for the students thus creating a mutual learning environment for all. In some cases, this was in terms of language acquisition on the students’ part, whereby the images and resources the students produced contained both Arabic and English spellings:

*‘We also learnt some Arabic which is really helpful. So, we know how to say “hello” and “my name is” so we are learning too.’* (SM)

However, for other students the learning was more abstract, with students referring to improved confidence, the need for relationship building and developing a reciprocal trusting relationship also being highlighted:

*‘I think it’s given me confidence as well, for when we do qualify, because although I think many of us have had some exposure to women who need interpretation, you can’t choose what exposure you get as students (SM).’*

*‘Kind of made me think and reflect on our practice, about how continuity models can be. Just because you can see that the relationship which has developed within what like 5 sessions … working with these women from day one to now, just in confidence alone they’ve grown, even if their English isn’t to a high standard, which obviously it’s not, but just about every one of them is able to communicate.’*(SM)

### Taking our learning forwards

The students wanted to improve the service that had been offered to the women and made numerous suggestions based on their placement experiences:

*‘I think it would be good to maybe have an “appointment” in English and then in Arabic. Then in English and Arabic “headache” and “blood” and “blood pressure”, just those normal observations that you’d get asked in any sort of healthcare setting. Those could be on flash cards because then we could ask a question and then use the flash cards, because I was using my phone to show pictures of things like blood and then I was getting a picture of blood and then saying “his is blood”.’* (SM)

The fact that the women have made progress in their English skills was an important benchmark for the students, leading them to value the placement from an impact perspective:

*‘It was actually really good, wasn’t it? Was really, really helpful. I think it’s something that we need to take forwards in placement and I actually spoke to the English tutor and asked how they did in the quiz, the test that they had last week. And I believe they all passed it, which is something to reflect on because obviously we’ve helped them, so I think that’s something for us all to take away. Really what we’ve all achieved.’* (SM)

*‘I really enjoyed it and I could see different learning going on so that we were learning how to communicate a little bit better and about having a bit more of that understanding of how difficult it can be.’* (SM)

The program also impacted on some of the students who appeared to exhibit a ‘new found empathy’ when reflecting on the challenges of being in a country where a person’s first language was not spoken:

*‘Made me think how vulnerable it can be in a country where it wasn’t your first language and you were having a baby and you couldn’t get across to the midwife even the simplest concerns of maybe having tender breasts or a sore back or any sort of nipple pain. You couldn’t. You wouldn’t be able to sort of explain that because of your language barrier. So, it made me feel … I wouldn’t like to give birth in a country where they couldn’t understand me well.’* (SM)

Being part of the program also opened students’ eyes to the challenges they may face in their future midwifery practice when caring for women who did not speak English:

*‘It showed what barriers that we’re gonna come up against and the struggles we’ll have.’* (SM)

## DISCUSSION

This study evaluated a non-traditional community placement where student midwives participated in English classes with women whose first language was Arabic. Using Kolb’s experiential learning model, findings show the placement to be a valuable learning experience for both students and the women who participated. The student midwives exhibited reflexive learning, acquiring the knowledge they could ‘tangibly’ transfer to their future midwifery practice. They also gained first-hand experience with the challenges of ‘educating’, which is key to the role of the midwife. In addition, the students demonstrated problem-solving skills and creativity when rising to the challenges the ESOL program placement presented them, adapting and personalizing their communication, something that is critical to effective midwifery care^21^.

There was a degree of expectation versus reality in terms of the student experiences from the placement, something that has been highlighted previously by authors in terms of student midwife placements^22,23^. Evidence shows students can meet the demands of their program negatively to positively^24,25^, with the latter being the case with the students in the current study. The nature of the placement, which allowed the students increased ‘agency’ in terms of delivering education, was probably a contributory factor, and it should be acknowledged that clinical practice and assessment were not at stake. However, this does not remove the value of the placement, particularly given that it was not with pregnant women, as first expected. The latest NMC (2021) requirement for all student midwives in the UK to be placed in ‘alternative maternity settings’^11^ seeks to expose students to differing experiences and practice. However, an opportunity may have been missed to broaden this out to non-maternity settings that expose students to meaningful socio-cultural experiences.

During the placement, it was evident that bi-directional learning was taking place, with both women and students engaging in a mutual learning environment. The women’s experiences in terms of being taught by the students were wholly positive. The students experienced first-hand the well-documented challenges that women whose first language is not English may experience when in the UK maternity care system^26,27^. In 2020 in the UK, women from black ethnic groups were four times more likely to die in pregnancy than women from white groups, and women from Asian ethnic backgrounds were almost twice as likely to die^28^. In a systematic review of the literature, Khan^29^ identified five key themes that impact women from ethnic minority groups’ experiences and use of health services: communication, midwife-woman relationship, healthcare services and systems, culture, and social needs. The student midwives gained an understanding of the importance of all of these areas during their placement, and although the placement did not set out to measure changes in student cultural awareness, one could conclude that this had increased.

The students described instances of placing themselves in the ‘women’s shoes’ and displayed a newfound recognition of the barriers the women faced. This echoes findings from the Prout et al.^30^ study, whereby health science students undertook a novel placement, ‘Country Week’, in rural Australia to gain an understanding of the challenges faced by residents within these communities. Prout et al.^30^ described how the students developed a strong understanding in terms of these challenges and how this could only be learned on placements which immersed students in such communities. One could argue that the level of cultural awareness the student midwives acquired could not be achieved to the same extent in a busy clinical area, where time is constrained, and the emphasis is on clinical rather than cultural competence.

There appeared to be a strong element of ‘self-efficacy’ in terms of student learning. This could be due to the setting the students were placed in, which appeared to cultivate a rich learning environment. This is comparable to the Folkvord and Risa^31^ qualitative systematic review of significant factors that engender self-efficacy in student midwives during clinical placements. They concluded that ‘A learning culture appears to be the fertile ground in which midwife students thrive’. The ESOL program placement also enabled students to experience continuity, purpose, achievement, and significance, elements of the key senses’ framework developed by Dewar et al.^32^ when exploring an optimum environment for student midwife learning.

### Limitations

This study is not without its limitations. Students volunteered to undertake the placement and, as such, had a vested interest in it; students undertaking it as a mandatory placement may not have experienced it as positively. The placement setting was unique and was overseen by a lecturer in practice placements, which could reduce the transferability of findings. Only three participants took part in the focus group, which limited the voices of the women the placement was hoping to benefit from.

## CONCLUSIONS

The shortage of midwives in the UK has led to an increase in training places being commissioned; however, this, in turn, places pressure on the existing midwifery workforce tasked to supervise and assess the students. This study demonstrates how placing student midwives in a unique non-maternity setting can lead to clear benefits in their learning. It also illustrates that small-scale placements can give valuable returns and, in this case, reciprocally contribute to initiatives aimed at reducing health inequalities in our communities. Importantly, it confirms that the quality of learning during a non-traditional placement is not compromised and that health students can gain valuable knowledge and skills that they may not otherwise achieve in traditional placements.

## Data Availability

The data supporting this research are available from the authors on reasonable request.
